# Rural-urban disparity in category II vaccination among children under five years of age: evidence from a survey in Shandong, China

**DOI:** 10.1186/s12939-018-0802-4

**Published:** 2018-06-22

**Authors:** Xinyi Zhang, Zerin Imam Syeda, Zhengyue Jing, Qiongqiong Xu, Long Sun, Lingzhong Xu, Chengchao Zhou

**Affiliations:** 1 School of Public Health, Shandong University, Jinan, 250012 China; 2 Key Lab of Health Economics and Policy Research, Shandong University, 44 Wen-hua-xi Road, Jinan, 250012 Shandong China

## Abstract

**Background:**

Compared with the Expanded Program on Immunization (EPI) vaccines, the coverage rate of the non-EPI vaccines is still low. The aim of this study is to explore the rural-urban disparity in category II vaccine and its determinants among children under 5 years old in China.

**Methods:**

A cross-sectional study was conducted in 17 cities in Shandong province from August to October, 2013. A total of 1638 children were included in the analysis. Unadjusted and adjusted regression model were used to identify the rural-urban difference in vaccination of category II vaccine. Multivariate logistic regression models were employed to analyze the determinants associated with vaccination of category II vaccine in rural and urban areas respectively.

**Results:**

The coverage rates of category II vaccine in rural and urban children were 81.5 and 69.4% respectively. Factors including age and satisfaction with vaccination services were associated with category II vaccination both in rural and urban children (*Ρ* < 0.05). It was also found that the households with four or less members are more likely to vaccinate category II vaccine in rural children.

**Conclusions:**

There was a big difference between rural and urban children in the use of category II vaccine. The government should strengthen financial support and regulation for the category II vaccine. The identified at-risk factors, including age, satisfaction with the vaccination services, and family size should be taken into account when designing targeted vaccination policies for rural and urban children.

## Background

Vaccine immunization is a highly cost-effective public health intervention which has played an important role in preventing children from infectious diseases [1]. According to the World Health Organization (WHO) estimates in 2012, immunization can prevent 2 to 3 million deaths each year [2]. Furthermore, among the most cost-effective measures to address the xpanded children’s immunization coverage ranked third, at the Copenhagen Consensuworld’s major challenges, es Center in 2012 [3]. China established the expanded program on immunization (EPI) in 1978. In 2007, China implement the national EPI, the type of vaccine-preventable diseases also expanded from 6 to15 [4]. The EPI vaccines are provided free of charge by the government, while non-EPI vaccine, which is also called category II vaccine or self-paid vaccine, is a voluntary vaccine by the citizens at their own expense [5].

Globally, vaccine-preventable disease is a serious public health burden [6]. China has a large birth cohort, so the category II vaccine such as H. Influenza type b (HIB) and S. pneumoniae etc. corresponding diseases are more worthy of attention. The WHO estimates that China’s S. pneumoniae infections and deaths accounted for 12 and 3.6% of the global burden, and HIB infections and deaths accounted for 14 and 5.1% of the global burden, respectively [7, 8]. Many studies have demonstrated that expanding coverage of category II vaccine has a highly cost-effectiveness [9–11].

However, in China, the actual vaccination rate of category II vaccines is remarkably different from those of EPI vaccines and inoculation rate varies widely in different regions. Previous studies have demonstrated that the coverage of category II vaccines in children aged 1~ 2 was 61.42% in China, while this proportion was 82.51, 64.86 and 43.18% in eastern, central and western China respectively [12].Other studies have also demonstrated that there was a difference in the utilization of category II vaccines among families with different socio-economic status [1, 13–15].

Most of the existing studies only focused on the associated factors and vaccination rates for category II vaccine [16–19].To date, no studies have examined the difference in the rates of category II vaccination in children between urban and rural China. In this study, we aim to explore the difference in the use of category II vaccine between rural and urban China. To do so, we have the following specific objectives. First, we will compare the use of category II vaccine between rural and urban children in China. Second, we will identify the factors associated with the use of category II vaccine in rural and urban children respectively.

## Methods

### Data source

Data used in this study were obtained from the 5th National Health Service Surveys (NHSS) of Shandong conducted in 2013. NHSS is a nationally representative survey of China organized and directed by the National Health and Family Planning Commission of China every 5 year. Shandong is the second largest province in China and its children (0–14) account for 16.1% of the total population (about 97million) [20].According to previous studies, category II vaccination rate among children under 5 years old was significantly lower than that of EPI vaccines [21, 22]. In this study, a four-stage, stratified cluster sampling was used to select participants. First, we randomly selected 20 counties (urban districts) from all prefectural cities in Shandong province, and in each county (district), we selected 5 townships (sub-districts). Second, we chose two villages (communities) from the each selected township (sub-district) randomly. Thus, 100 township (sub-districts) and 200 villages (communities) were chosen as study sites. Third, 60 children were randomly selected from each sample village (community) and the youngest one in each household was investigated. Eventually, a total of 1638 children were included in the analysis, of which the number of urban children was 764, and rural children was 874.

### Variables and measures

#### Dependent variable

The dependent variable was vaccination of category II vaccines in children under 5 years old, which was assessed on the ground of primary guardians’ answers to – “Has your child been vaccinated with the following category II or self-paid vaccines (Haemophilus influenzae type b vaccine, Pneumococcal polysaccharide vaccine, Varicella attenuated live vaccine, Influenza vaccine and Measles and mumps combined attenuated live vaccine or others)?” As long as vaccinate any one type or more of the category II vaccines, it was classified as “yes”. The difference between category I and category II vaccines lies in that the category I vaccines are provided free of charge by the government, but the category II vaccines are paid fully by the users themselves. When conducting the interview, the vaccination certificate of the interviewed child would be also reviewed by the interviewers to confirm the response of the interviewees.

#### Independent variable

Socio-demographic characteristics, which including gender (boy versus girl), age (0–1, − 2, − 3, − 4, − 5 years), family size (≤ 4 versus>4), primary caregiver (grandparents versus parents), primary caregivers’ education attainment (primary and below, junior school, senior school and above), physical examination (yes vs. no), satisfaction (satisfaction vs. non-satisfaction), and household income (Q1, Q2, Q3, Q4 and Q5).

“Physical examination” was collected by asking the children’s primary caregivers “How many times physical examinations have your child had received in the past 12 months (Excluding those examinations for the diagnosis or treatment for some special diseases)?” If the answer was “once or more”, we coded it as “yes”; and if the answer was “none”, we coded it as “no”.

As for “Satisfaction”, we collected the data by asking the primary caregivers a question of “Are you satisfied with the vaccination services the health care facilities provide for your child?” . If the answer was “yes”, we coded it as “yes”; and if the answer were “normal” or “no”, we coded it as “no”.

When measuring “household income”, we used a question of “How much was the total income of your family in the last year? (Urban households were disposable income, rural households were net income)” It was divided into the same group distance: 20, 40, 60, and 80%, based on the total income of households in the last year from highest to lowest, the income quintiles can better reflect the grouping of resident’s income. Quintile 1 (Q1) was the poorest and Quintile 5 (Q5) was the richest.

#### Statistical analysis

The data were double entered using EpiData 6.04. Statistical analyses were performed using SPSS 16.0. Chi-square tests were employed to evaluate socio-demographic differences between urban and rural children. Two models, one unadjusted and one adjusted regression model were used to identify the rural-urban difference in vaccination of category II vaccines. Multivariate logistic regression model was employed to analyze relevant factors associated with vaccination of category II vaccines in rural and urban areas respectively. Sampling weights were included in all analyses to adjust for the complex survey design. Statistical significance was set at the 5% level.

## Results

### Socio-demographic characteristics

A total of 1638 children and their primary caregivers participated in this study, of which urban children accounted for 46.6% (764), and rural children accounted for 53.4% (874). There were no significant differences in gender, age, family size, physical examination, and satisfaction between urban and rural children. Generally speaking, the probability of urban children lived in higher-income households as higher than that in rural children (*Ρ*= 0.000). Among the primary caregivers of urban children, the proportion of parents is higher (*Ρ*= 0.000) than that of rural children, and the primary caregivers also had significant higher education attainment in urban children (*Ρ*= 0.000) than that in rural children (Table 1).

**Table 1:** Socio-demographic characteristics of study participants in Shandong, China, 2013

Variable	Overall	Urban	Rural	^*^P - value
N (%)	1638(100)	764	874	
Sex				0.136
Boy	894(54.6)	402(52.6)	492(56.3)	
Girl	744(45.4)	362(47.4)	382(43.7)	
Age				0.714
0–1	500(30.5)	240(31.4)	260(29.7)	
-2	320(19.5)	149(19.5)	171(19.6)	
-3	350(21.4)	169(22.1)	181(20.7)	
-4	303(18.5)	132(17.3)	171(19.6)	
-5	165(10.1)	74(9.7%)	91(10.4)	
Family size				0.241
≤ 4	916(55.9)	439(57.5)	477(54.6)	
> 4	722(44.1)	325(42.5)	397(45.4)	
Household income ^a^				*0.000*
Q1	363(22.2)	121(15.8)	242(27.7)	
Q2	292(17.8)	117(15.4)	175(20.0)	
Q3	333(20.3)	149(19.5)	184(21.1)	
Q4	364(22.2)	186(24.3)	178(20.4)	
Q5	286(17.5)	191(25.0)	95(10.9)	
Physical examination				0.320
Yes	988(60.3)	451(59.0)	537(61.4)	
No	650(39.7)	313(41.0)	337(28.6)	
Primary caregiver				*0.000*
Parents	1445(88.2)	700(91.6)	745(85.2)	
Grandparents	193(11.8)	64(8.4)	129(14.8)	
Primary caregiver’s education				*0.000*
Primary and below	247(15.1)	44(5.8)	203(23.2)	
Junior education	821(50.1)	320(41.9)	501(57.3)	
Senior and above	570(34.8)	400(52.4)	170(19.5)	
Satisfaction ^b^				0.496
Satisfaction	1396(85.2)	656(85.9)	740(84.7)	
Non-satisfaction	242(14.8)	108(14.1)	134(15.3)	

Note: The *P*-values indicate statistical significance at 5% level, the italics indicate significance

^a^Quintile 1(Q1) is the poorest and Quintile 5(Q5) is the richest *: Chi-test

^b^Satisfaction with the vaccination services

### Category II vaccination rate

**Fig. 1:**
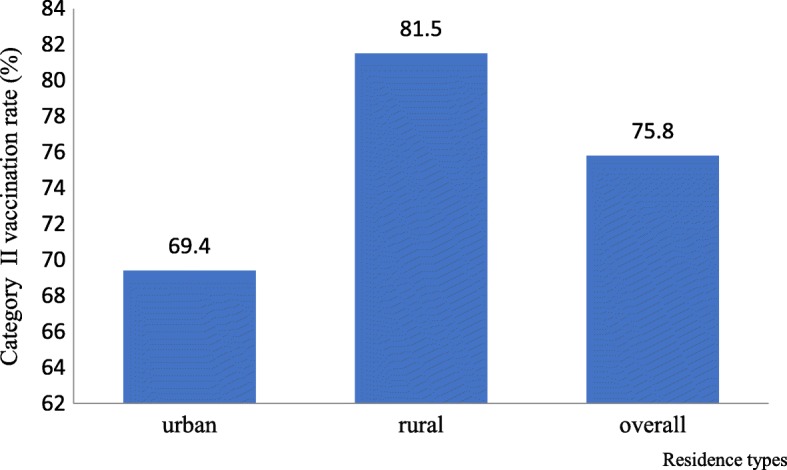
Category II vaccination rate of children among different groups in Shandong, China, 2013

Category II vaccination rate of children among different groups is shown in Fig. 1. We can clearly see that the overall rate of category II vaccines was 75.8% (1242/1638). When we compared the category II vaccination rate between urban and rural children, we found that the rate of category II vaccination was significantly higher in rural children (81.5%) than that in urban children (69.4%).

### Category II vaccination status in urban and rural areas

We used two models, one unadjusted and one adjusted logistic regression model, to assess the association between residence and category II vaccination status in children under 5 years old (Table 2). The unadjusted model showed that the rate of category II vaccines in rural children was statistically higher than that in urban children (*Ρ*= 0.000). When we controlled for other variables, compared with urban children, vaccination rate was still higher in rural children (*Ρ*= 0.000).

**Table 2:** Association of category II vaccination status with residence types in Shandong, China

Variable	Model 1 (No covariates)			Model 2 (covariates)		
	Ρ	OR	OR95%CI	P	OR	OR95%CI
Area						
Urban		1.0			1.0	
Rural	*0.000*	1.94	1.54–2.44	*0.000*	1.93	1.48–2.52
Sex						
Boy					1.0	
Girl				0.829	1.03	0.81–1.31
Age						
-5					1.0	
0–1				*0.000*	0.29	0.18–0.46
-2				0.575	0.87	0.52–1.43
-3				0.457	1.22	0.73–2.04
-4				0.738	1.09	0.65–1.84
Family size						
> 4					1.0	
≤ 4				0.443	1.10	0.86–1.41
Household income						
Q5^a^					1.0	
Q1				*0.026*	0.64	0.43–0.95
Q2				0.448	0.86	0.57–1.31
Q3				0.339	1.23	0.81–1.86
Q4				0.861	0.97	0.66–1.42
Physical examination						
No					1.0	
Yes				0.479	1.09	0.85–1.41
Primary caregiver						
Grandparents					1.0	
Parents				0.497	1.14	0.85–1.41
Primary caregiver’s education						
Primary and below					1.0	
Junior education				0.202	1.28	0.88–1.88
Senior and above				0.411	0.84	0.55–1.27
Satisfaction ^b^						
Satisfaction					1.0	
Non-satisfaction				*0.000*	1.87	1.37–2.59

Note: The P-values indicate statistical significance at 5% level, the italics indicate significance

^a^Quintile 1(Q1) is the poorest and Quintile 5(Q5) is the richest

^b^Satisfaction with the vaccination services

### Determinants of category II vaccination status among urban and rural children

Univariate analysis was conducted to identify the factors independently associated with category II vaccination status in rural children (Table 3) and urban children (Table 4) respectively. Two factors were found to be determinants (*P < 0.05*) both in rural and urban children, including age and satisfaction. Compared with urban children, children in rural areas which with less than four family members tended to use category II vaccines (*P < 0.05*). We also observed the same results in multivariate logistic regression models for the rural and urban children (*P < 0.05*).

**Table 3:** Factors associated with category II vaccination status of children in urban Shandong, China, 2013

Variable	Vaccinated (%)	Unvaccinated (%)	P	ORc^*^	ORc95%CI	P	ORa^*^	ORa95%CI
*n* = 764	530(69.4)	234(30.6)						
Sex						NA^*^		
Boy	279(69.4)	123(30.6)		1.0				
Girl	251(69.3)	111(30.7)	0.984	0.99	0.73–1.36			
Age								
-5	60(81.1)	14(18.9)		1.0			1.0	
0–1	111(46.3)	129(53.7)	*0.000*	0.20	0.11–0.38	*0.000*	0.21	0.11–0.39
-2	114(76.5)	35(23.5)	0.438	0.76	0.38–1.52	0.485	0.78	0.38–1.58
-3	135(79.9)	34(20.1)	0.829	0.93	0.46–1.85	0.873	0.94	0.47–1.92
-4	110(83.3)	22(16.7)	0.683	1.17	0.56–2.45	0.433	1.36	0.63–2.90
Family size						NA		
> 4	232(71.4)	93(38.6)		1.0				
≤ 4	298(67.9)	141(32.1)	0.299	0.85	0.62–1.16			
Household income						NA		
Q5^a^	136(71.2)	55(28.8)		1.0				
Q1	79(65.3)	42(34.7)	0.272	0.76	0.47–1.24			
Q2	78(66.7)	39(33.3)	0.402	0.81	0.49–1.33			
Q3	115(77.2)	34(22.8)	0.214	1.73	0.83–2.24			
Q4	122(66.6)	64(33.4)	0.242	0.77	0.50–1.19			
Physical examination						NA		
No	225(71.9)	88(28.1)		1.0				
Yes	305(67.6)	146(32.4)	0.210	0.82	0.59–1.12			
Primary caregiver						NA		
Grandparents	42(65.6)	22(34.4)		1.0				
Parents	488(69.7)	212(30.3)	0.497	1.21	0.70–2.07			
Satisfaction ^b^								
Satisfaction	62(57.4)	46(42.6)		1.0			1.0	
Non-satisfaction	468(71.3)	188(28.7)	*0.004*	1.85	1.22–2.81	*0.003*	2.02	1..26–3.22
Primary caregiver’s education attainment						NA		
Primary and below	32(72.7)	12(27.3)		1.0				
Junior education	242(75.6)	78(24.4)	0.676	1.16	0.57–2.37			
Senior and above	256(64)	144(36)	0.252	0.67	0.33–1.34			

Note: The P-values indicate statistical significance at 5% level, the italics indicate significance

^*^ORc Crude odds ratio, ^*^ORa Adjusted odds ratio, ^*^NA Not applicable

^a^Quintile 1(Q1) is the poorest and Quintile 5(Q5) is the richest

^b^Satisfaction with the vaccination services

**Table 4:** Factors associated with category II vaccination status of children in rural Shandong, China, 2013

Variable	Vaccinated (%)	Unvaccinated (%)	P	ORc^*^	ORc95%CI	P	ORa^*^	ORa95%CI
*n* = 874	712(81.5)	162(18.5)						
Sex						NA^*^		
Boy	400(81.3)	92(18.7)		1.0				
Girl	312(81.7)	70(18.3)	0.888	1.03	0.73–1.45			
Age								
-5	77(84.6)	14(15.4)		1.0			1.0	
0–1	184(70.8)	76(29.2)	*0.011*	0.44	0.24–0.83	*0.003*	0.36	0.19–0.71
-2	144(84.2)	27(15.8)	0.932	0.97	0.48–1.96	0.681	0.86	0.42–1.77
-3	164(90.6)	17(9.4)	0.146	1.75	0.82–3.74	0.285	1.53	0.71–3.32
-4	143(83.6)	28(16.4)	0.835	0.93	0.46–1.87	0.632	0.84	0.41–1.73
Family size								
> 4	311(78.3)	86(21.7)		1.0			1.0	
≤ 4	401(84.1)	76(15.9)	*0.031*	1.46	1.04–2.06	*0.010*	1.62	1.12–2.33
Household income						NA		
Q5^a^	77(81.1)	18(18.9)		1.0				
Q1	181(74.8)	61(25.2)	0.224	0.69	0.39–1.25			
Q2	143(81.7)	32(18.3)	0.894	1.05	0.55–1.98			
Q3	157(85.3)	27(14.7)	0.359	1.36	0.71–2.62			
Q4	154(86.5)	24(13.5)	0.235	1.50	0.77–2.93			
Physical examination						NA		
No	269(79.8)	68(20.2)		1.0				
Yes	443(82.5)	94(17.5)	0.323	1.19	0.84–1.69			
Primary caregiver						NA		
Grandparents	105(81.4)	24(18.6)		1.0				
Parents	607(81.5)	138(18.5)	0.983	1.01	0.62–1.63			
Satisfaction ^b^						NA		
Satisfaction	96(71.6)	38(28.4)		1.0			1.0	
Non-satisfaction	616(83.2)	124(16.8)	*0.002*	1.97	1.29–3.00	*0.007*	1.87	1.19–2.93
Primary caregiver’s education attainment						NA		
Primary and below	162(79.8)	41(10.2)		1.0				
Junior education	416(83.0)	85(17.0)	0.312	1.24	0.53–1.22			
Senior and above	134(78.8)	36(21.2)	0.816	0.94	0.57–1.56			

Note: The P-values indicate statistical significance at 5% level, the italics indicate significance

^*^ORc Crude odds ratio, ^*^ORa Adjusted odds ratio, ^*^NA Not applicable

^a^Quintile 1(Q1) is the poorest and Quintile 5(Q5) is the richest

^b^Satisfaction with the vaccination services

## Discussion

The present study explores, for the first time, the rural-urban difference in category II vaccination of children under 5 years in China. This study shows that the overall rate of category II vaccines for children aged 0–5 is 75.8% in Shandong province. The rate of category II vaccines is lower than the 88.24% found in Jiangdong district, China [23]. It’s also a little lower than the rate of 78.31% in the children aged 2 to 6 years in Tianjin [24]. However, it is higher than the inoculation rate of category II vaccines (61.42, 73.24%) in some other studies in China [12, 25]. Compared with economically developed regions in China, there is still a little gap in the category II vaccination rate in Shandong province.

Contrary to previous studies in China [17, 26], our results show that category II vaccines rate in urban children is significantly lower than that in rural children. Different from the unified national policy for the EPI vaccines, the current provision of category II vaccines mainly depends on the grassroots’ disease prevention and control units and even vaccination stations [27]. In rural areas, primary health providers usually have close connection with residents. Therefore, the primary caregivers of rural children are better encouraged to vaccinate, with the efforts of rural health providers [28].Secondly, it is found in previous studies that perceived social norms were positively correlated with willingness to pay [29]. The social impact of the group effect is an important factor in the decision-making of the child’s vaccination. This group effect is more obvious in the rural areas where the population is closely linked. This is also consistent with the findings in other countries [30–32].Thirdly, vaccination safety has always been a concern of the guardians, and any negative news of vaccination is likely to cause short or long-term effects on vaccination use. Many guardians are afraid of the vaccination insecurity or fear of adverse reactions, as a result they would not use vaccination [27, 33]. In the rural areas, where the influence of mass media is limited, this negative effect will be relatively low [34]. The above reasons might explain the rural-urban difference in category II vaccination use found in the current study.

Economic status was found to be a determinant for category II vaccines use, which was similar with previous studies [17–19]. Ideally, the health service system should ensure equal access to public health services, regardless of gender, residence and capacity to pay. In the current study, compared with high-income households, lower income ones have a lower probability of vaccination. One possible reason is that vaccine pricing and funding mechanisms have a significant impact on children’s vaccination. Currently, there is no uniform pricing standard for category II vaccines in China. This provides the possibility for healthcare providers to raise the price of the vaccines. The study conducted in 2013 on the willingness of category II vaccines indicated that the procurement price up to 15 and 43% for county CDC (Center for Disease Prevention and Control) and vaccination clinics, respectively [35], and this price elasticity would probably affect the demand for vaccination, especially in low-income households. Another possible reason might be that, unlike the EPI vaccines with the government financial subsidies, the cost of the category II vaccine is entirely paid by the vaccine recipients. Such cost, in most cases, might adversely affect the use of category II vaccines. This finding indicates a need for the government to regulate the management of category II vaccines, so as to ensure the rationality and transparency of vaccine prices. In addition, financing subsidies should also be expanded to reduce the cost of category II vaccines inoculation, especially for those low-income households.

Among the socio-economic factors, age and satisfaction for the vaccination services are significantly associated with the category II vaccination use both in rural and urban children. Our study demonstrates that those children, who are older, whose primary caregivers are satisfied with immunization institutions’ service are more likely to vaccinate category II vaccines. Many previous studies have found that vaccination service providers play an important role in caregivers’ vaccination decisions, and caregivers’ satisfaction with vaccination is positively related to immunization [36, 37]. The results also show that the households with four or less members are more likely to vaccinate category II vaccines in rural children. One possible explanation might be that the number of children in a small family may be less, and in such families the caregivers might have more money to use category II vaccines for their children, compared with those families with more children [38–40].

Interestingly, the current study shows that there is no gender difference in vaccination rate both in rural and urban, which has also been found in previous studies [41, 42]. This might be due to that gender discrimination has never been a problem in China. Consistent with previous studies [26, 40], our study also shows that primary caregiver’s education attainment is not associated with vaccination rate.

This study had some limitations. First, the data used in this study were derived from a cross-sectional design, the associations between identified factors and category II vaccination cannot be interpreted as cause and effect. Second, many of the variables in our questionnaire were measured by self-reported, which might result in several kinds of bias, such as recall bias.

## Conclusions

This study finds that there is a significant gap in category II vaccine use in children under 5 years between urban (69.4%) and rural (81.5%) China. The factors including age and satisfaction for the vaccination services are found to be associated with category II vaccination use both in rural and urban areas. In addition, economic status is still a determinant for category II vaccine. The government should strengthen financial support and regulation for the category II vaccine. The identified at-risk factors, including age, satisfaction with the vaccination services, and family size should be taken into account when designing targeted vaccination policies for rural and urban children.

## Abbreviations

EPI: Expanded program on immunization;
HIB: H. influenzae type b.
NHSS: National Health Service Surveys
